# Genotype-Phenotype Relationships and Endocrine Findings in Prader-Willi Syndrome

**DOI:** 10.3389/fendo.2019.00864

**Published:** 2019-12-13

**Authors:** Régis Afonso Costa, Igor Ribeiro Ferreira, Hiago Azevedo Cintra, Leonardo Henrique Ferreira Gomes, Letícia da Cunha Guida

**Affiliations:** Laboratório de Alta Complexidade, Instituto Nacional da Saúde da Mulher, da Criança e Do Adolescente Fernandes Figueira, Fiocruz, Rio de Janeiro, Brazil

**Keywords:** Prader-Willi syndrome, genotype, phenotype, endocrine, imprinting, SNORDs

## Abstract

Prader-Willi syndrome (PWS) is a complex imprinting disorder related to genomic errors that inactivate paternally-inherited genes on chromosome 15q11-q13 with severe implications on endocrine, cognitive and neurologic systems, metabolism, and behavior. The absence of expression of one or more genes at the PWS critical region contributes to different phenotypes. There are three molecular mechanisms of occurrence: paternal deletion of the 15q11-q13 region; maternal uniparental disomy 15; or imprinting defects. Although there is a clinical diagnostic consensus criteria, DNA methylation status must be confirmed through genetic testing. The endocrine system can be the most affected in PWS, and growth hormone replacement therapy provides improvement in growth, body composition, and behavioral and physical attributes. A key feature of the syndrome is the hypothalamic dysfunction that may be the basis of several endocrine symptoms. Clinical and molecular complexity in PWS enhances the importance of genetic diagnosis in therapeutic definition and genetic counseling. So far, no single gene mutation has been described to contribute to this genetic disorder or related to any exclusive symptoms. Here we proposed to review individually disrupted genes within the PWS critical region and their reported clinical phenotypes related to the syndrome. While genes such as *MKRN3, MAGEL2, NDN*, or *SNORD115* do not address the full spectrum of PWS symptoms and are less likely to have causal implications in PWS major clinical signs, *SNORD116* has emerged as a critical, and possibly, a determinant candidate in PWS, in the recent years. Besides that, the understanding of the biology of the PWS SNORD genes is fairly low at the present. These non-coding RNAs exhibit all the hallmarks of RNA methylation guides and can be incorporated into ribonucleoprotein complexes with possible hypothalamic and endocrine functions. Also, DNA conservation between SNORD sequences across placental mammals strongly suggests that they have a functional role as RNA entities on an evolutionary basis. The broad clinical spectrum observed in PWS and the absence of a clear genotype-phenotype specific correlation imply that the numerous genes involved in the syndrome have an additive deleterious effect on different phenotypes when deficiently expressed.

## Introduction

Prader-Willi syndrome (PWS; OMIM 176270) was first described in 1956 by Andrea Prader, Alexis Labhart and Heinrich Willi based on a study of nine children with a common clinical tetrad: short stature, intellectual disability, obesity, and small hands and feet (1, 2). The phenotypic analysis was expanded in the following years and decades, revealing the complexity of the syndrome, affecting endocrine, cognitive and neurologic systems, metabolism, and behavior. PWS was the first human disease to be related to genomic imprinting errors, and also the first one shown to be caused by uniparental disomy (3, 4). This rare genetic disorder has a prevalence of 1 in 10,000–30,000 live births, males and females are affected equally in all ethnic groups (5).

The PWS critical region on chromosome 15q11-q13 is monoallelically expressed by paternally inherited genes, exclusively. The absence of expression of one or more of these genes contributes to different phenotypes of PWS (6, 7) and there are three main mechanisms of occurrence: paternal deletion of the 15q11-q13 region; maternal uniparental disomy 15; or imprinting defects (8–10). On the other hand, in the same region, the loss of expression of the *UBE3A* gene (preferentially maternally expressed) drives to Angelman syndrome, with completely different clinical characteristics. By their common implicated region and mechanisms, both syndromes are considered sister imprinted disorders (11, 12).

Clinical manifestations vary with age, impacting multiple body systems (Table 1). Fetal size is usually within the normal range. Compared to unaffected siblings, birth weight and body mass index (BMI) are 15% lower on average. Prenatal hypotonia may cause decreased fetal movement, abnormal fetal position at delivery, and increased incidence of assisted delivery or cesarean section (14, 15).

**Table 1:** Clinical characteristics and the nutritional phases in PWS.

**Median ages**	**Clinical characteristics**
Prenatal—birth	Decreased fetal movements
	Lower birth weight and body mass compared to sibs
0–9 months	Severe hypotonia
	Feeding problems and failure to thrive
9–25 months	Improved feeding and appetite
	Normal growth
	Delayed physical and social milestones
2.1–4.5 years	Weight increasing without appetite increase or excess calories
4.5–8 years	Weight increasing with appetite increase
	Global developmental delay
8 years—adulthood	Hyperphagic, rarely feels satiety
	Mild intellectual disability and behavior problems
	Hypogonadism
Adulthood	Appetite no longer insatiable for some
	Short stature and small hands and feet

*Gunay-Aygun et al. (13); Miller et al. (14); Driscoll et al. (5)*.

Severe hypotonia is a clinical hallmark of PWS, leading to failure to thrive during infancy due to lethargy and poor suck. Other common neonatal findings are decreased movement and spontaneous arousal, weak cry, thick saliva, and poor reflexes (13, 16). Around 9 months of life, eating behavior starts to normalize, and the hypotonic status tends to improve, but mild-to-moderate hypotonia persists throughout life, with reduced muscle mass and tone (9, 17).

Physical and social milestones (as sitting, walking, first words, and reading) are delayed and can be achieved at about double the normal age (18). Most individuals have mild intellectual disability, learning difficulties, and poor academic performance. During early infancy, characteristic behavioral problems are common, such as stubbornness, manipulation, compulsiveness, self-injury, and difficulty with change in routine (5, 19, 20). Another common feature in the syndrome is sleep disruption, related to sleep apnea that impairs the quality and efficiency of sleep, frequently associated with excessive daytime sleepiness, and sedentary behavior with a higher predisposition to obesity (13, 21).

In later childhood, individuals with PWS will reach severe obesity unless food intake is strictly controlled by family and caretakers. The lack of satiety (hypothalamic origin) results in hyperphagia, with obsessive food seeking. In uncontrolled cases, obesity, and its complications are the major causes of morbidity and mortality: respiratory insufficiency, cardiovascular problems, metabolic syndrome, sleep apnea, and type 2 diabetes mellitus (22, 23). Mortality rates range between 1.25 and 3% per year (24, 25). Hyperphagia in PWS is still not fully understood and controlling appetite remains a challenge.

The endocrine system can be the most affected in PWS. Growth hormone (GH) deficiency is present in up to 74% of cases and is associated with short stature, small hands and feet, low motor strength, increased fat mass, and decreased movement and energy expenditure (26, 27). GH replacement therapy has shown positive effects not only on growth and body composition but also on development, behavior, and nocturnal respiratory abnormalities, although a careful respiratory follow up is mandatory during long-term GH administration (28–34). Hypogonadism affects both sexes and is manifested as hypogenitalism, incomplete pubertal development and infertility in most individuals (35). Hypogonadism is thought to have a hypothalamic origin, and subsequent insufficient secretion of pituitary gonadotropins and sexual hormones (testosterone or estrogen) (7, 36, 37). Other endocrine abnormalities include hypothyroidism (20–30%), central adrenal insufficiency (about 5%) and type 2 diabetes (up to 25%) due to obesity complications (24, 38–41).

## Molecular Genetics and Diagnostic

**Figure 1:**
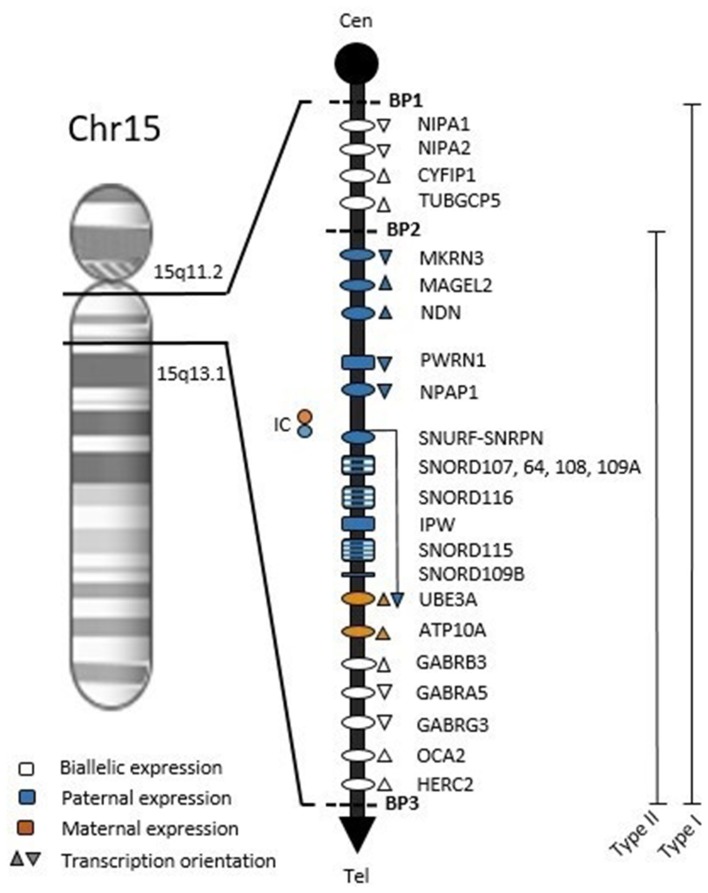
Chromosome map of 15q11.2-q13.1 region. Symbols: ovals, protein-coding genes; rectangles, RNA genes; BP1, breakpoint 1; BP2, breakpoint 2; BP3, breakpoint 3; Type 1, BP1-BP3 deletion with ~6 Mb; Type 2, BP2-BP3 deletion with ~5.3 Mb; Cen, Centromere; Tel, Telomere; IC, Imprinting Center.

The hypothalamic dysfunction observed in PWS may be the basis of several symptoms (such as hypotonia, developmental delay or obesity) that overlaps features of other conditions on clinical grounds, like normal obesity and intellectual disability (42). Definitive diagnosis requires DNA testing. The PWS region spans ~6 Mb on the long arm of chromosome 15 (Figure 1). Within this region, at least 2.5 Mb comprises genes with differential expression depending on parental origin. This locus holds protein-coding genes and several non-coding RNAs, which are believed to be involved in the regulation of alternative splicing, mainly in the brain (10, 16).

The bicistronic gene *SNURF-SNRPN* is central to the PWS region and crucial to understanding the methylation pattern in the syndrome. The CpG island at the 5′ end of *SNURF-SNRPN* (encompassing the promoter region, exon 1 and intron 1) is differentially imprinted according to parental origin: the unmethylated paternal allele is expressed while the methylated maternal allele is repressed (43). The PWS imprinting center (PWS-IC, Figure 1) involves the CpG island and exon 1 within the 4.3 Kb smallest region of overlap (44). Furthermore, *SNURF-SNRPN* expression produces a long transcript also including PWS-IC, Six snoRNA genes, *IPW* and *UBE3A* antisense (Figure 1), which is hypothesized to repress paternal *UBE3A* (45–48).

Most PWS patients (65–75%) present a 5–6 Mb deletion at 15q11-q13 from the paternal origin (16, 49). There are two proximal breakpoints and a common distal breakpoint (Figure 1), these regions are flanked by low copy repeat sequences that predispose to abnormal chromosomal pairing and uneven crossing-over, resulting in errors during meiosis (50, 51). Maternal Uniparental Disomy (mDUP) occurs when both chromosomes 15 are inherited from the mother and accounts for ~20–30% of cases, being associated with advanced maternal age (9, 15). Imprinting defects are caused by epimutations or microdeletions in the PWS-IC in 1–3% of PWS cases. These individuals have biparental allele inheritance, but a maternal-only DNA methylation pattern (11, 52).

Clinical and molecular complexity in PWS enhances the importance of genetic diagnosis in therapeutic definition and genetic counseling. Only DNA methylation analysis can consistently diagnose the syndrome in all three molecular classes (deletion, mUPD, and imprinting defects) and differentiate it from Angelman Syndrome (9, 52). The methylation analysis targets the 5′ CpG island of the *SNURF-SNRPN* locus and will correctly diagnose more than 99% of cases. Currently, there are three assays with this detection capacity: methylation-specific PCR (MS-PCR, the gold standard), methylation-specific multiplex ligation-dependent probe amplification (MS-MLPA) and methylation-sensitive high-resolution melting (MS-HRM) (53–58).

After methylation status confirmation, defining the exact molecular mechanism behind the syndrome origin is important for genetic counseling. Sporadic deletion cases have <1% risk of recurrence, while rare cases of structural abnormalities involving chromosome 15 (such as translocations, ring formation, isochromosome or inversions) can be as high as 25–50% and fluorescence *in situ* hybridization (FISH) can address the deletion source (59–65). mUPD 15 is typically *de novo* (recurrence <1%), proband and parents should be investigated by small nucleotide polymorphisms (SNP) microarray for accurate counseling (66, 67). Most imprinting defects cases are classified as epimutations with no alteration in the DNA sequence and have <1% recurrence risk. However, 15% of individuals with imprinting defects present a paternally inherited microdeletion (7.5–100 kb) in the PWS-IC, in which cases the risk of recurrence is 50%. IC analysis by MS-MLPA or DNA sequencing will address the exact origin of this event (5, 44).

## Genotype-Phenotype Relationships in Prader-Willi Syndrome

None of the PWS genetic errors are associated with exclusive symptoms. However, the most prevalent molecular classes (deletion and mUPD) show statistical differences in frequency or severity in some clinical characteristics. Patients with paternal deletion were more related to feeding problems, sleep disturbances, hypopigmentation and speech and language deficits (68). Individuals with the larger type 1 deletion (Figure 1) have been reported to have better academic performance and intellectual abilities, and more compulsiveness when compared to type 2 deletion patients (69, 70). Several other features are more common in mUPD individuals, such as post-term delivery, higher verbal IQ, psychosis and autism spectrum disorder (15, 69, 71–75). On the other hand, mUPD patients are less likely to have the typical PWS facial appearance or hypopigmentation (16). So far, no single gene mutation has been described to contribute to this genetic disorder. Here we proposed to review genes individually disrupted within the PWS critical region and their reported clinical phenotypes related to the syndrome.

The Makorin Ring Finger Protein 3 (*MKRN3, ZNF127*) gene encodes a zinc finger protein of the Makorin family and is paternally expressed ubiquitously in human adult tissues, with the highest level in testis, although its exact mechanism of action remains to be elucidated (76). This gene is associated with inhibition of puberty initiation, and loss of function mutations in *MKRN3* are recognized as the main genetic cause of Central Precocious Puberty (77). This correlation has been described by distinct studies with different ethnic groups, affecting equally both sexes, with all mutations segregated in a paternal manner at *MKRN3* (78–81). Experimental models with mice also support the correlation between mutations in *Mkrn3* and puberty dysfunctions, suggesting it may play a role in the hypothalamic-pituitary-gonadal axis (77–79). Altogether, this makes *MKRN3* a strong candidate gene for hypogonadism and infertility in PWS.

The physiological consequence of loss of expression of MAGE Family Member L2 (*MAGEL2*) has been related to phenotypic characteristics of PWS (82). *Magel2*-null mice exhibited endocrine dysfunction similar to PWS: neonatal growth retardation; excessive weight gain; increased adiposity after weaning; impaired hypothalamic regulation and changes in circadian rhythm (83, 84). Hyperphagia, commonly observed in individuals with PWS, is associated with a defect in the hypothalamic arcuate nucleus, which is the major action site of multiple complex interactions between neuropeptide Y (NPY), agouti-related peptide (AgRP), proopiomelanocortin (POMC), and leptin, regulating the food intake and body weight (85, 86). NPY/AgRP interaction stimulates food intake, whereas POMC reduces it. Loss of *MAGEL2* expression disturbs leptin-mediated depolarization of POMC neurons, indicating that food intake is being less repressed and fat storage regulated by leptin is uncontrolled (87, 88). Additionally, loss of expression of *Magel2* impairs reproductive function in mice. *Magel2*-null females showed extended and irregular estrous cycles, while males displayed decreased testosterone levels, and reduced pheromone detection, which has a direct relationship between the main olfactory epithelium and the hypothalamic GnRH neuronal system (89, 90). These results suggest that lack of expression of *MAGEL2* contributes to the reproductive deficiencies observed in PWS and also highlights the role of normal circadian rhythm in maintaining fertility.

Therein, specific point mutations on the paternal allele of *MAGEL2* were reported in 4 individuals with PWS spectrum phenotype: muscle hypotonia, weight gain, developmental delay, and hypogonadism. Although all clinical characteristics were consistent with PWS clinical diagnosis, methylation analysis on the promoter-exon 1 region of the *SNURF-SNRPN* gene showed normal allelic patterns (82). All four subjects were diagnosed with an autism spectrum disorder, intellectual disability, and different degrees of clinical and behavioral features of PWS. Although not a main characteristic, autism is present in 19% of individuals with PWS (71). These four individuals presented a normal methylation pattern, not compatible with PWS, despite similar clinical conditions, which was subsequently called Schaaf-Yang Syndrome (SYS) (91). Recent data of an international cohort of 78 patients with truncating *MAGEL2* mutations emphasized that SYS overlaps with PWS on clinical grounds in the early stages of life but diverges with the advance of childhood and adolescence (92). PWS features such as hypopigmentation, facial appearance, small hands and feet, thick saliva, behavioral problems are not commonly seen in SYS. And above all distinct symptoms, SYS does not usually cause the high appetite and severe obesity observed in PWS, which can disassociate *MAGEL2* and the hyperphagia condition.

The Necdin (*NDN*) gene encodes a DNA binding protein highly expressed in mature hypothalamic neurons (93). It has been postulated as a key regulator of GnRH levels both *in vitro* and *in vivo*, modulating essential intracellular processes for neurite and axonal outgrowth (94–96). Lack of *NDN* reduces GnRH gene expression, leads to decreased numbers of GnRH neurons, and decreased targeting of GnRH axons to the median eminence of the hypothalamus during development, which can contribute to hypogonadism and infertility in PWS. Also, Necdin paternal-deficient mice were associated with alterations in serotonin and respiratory systems, resulting in irregular breathing and sleep apneas, commonly observed in PWS. Another important evidence reported with *Ndn*-KO mice was sudden death due to respiratory disorders, which is the main side effect associated with GH therapy (97–99). *NDN* might be a genetic factor contributing to apneas and respiratory dysfunctions of PWS.

Interestingly, (100) described three patients with atypical deletions related to PWS. Patient 1 was deleted for *MKRN3, MAGEL2*, and *NDN* with no PWS major clinical criteria, except for obesity, developmental delay, and high pain threshold. Patients 2 and 3 had a deletion encompassing *NPAP1, SNURF-SNRPN*, and the SNORD genes, but did not reach *MKRN3, MAGEL2*, and *NDN*, and presented PWS major clinical signs (100). This report suggests that a paternal deficiency of *MKRN3, MAGEL2*, and *NDN* is not sufficient to generate the full PWS phenotype and postulates *NPAP1, SNURF-SNRPN*, and the SNORD genes (discussed ahead) to be the critical region for PWS. These results contradict other studies and exemplify the complexity to establish a genotype-phenotype relationship in PWS (78, 82, 83, 98, 99, 101, 102).

The Prader-Willi region encompasses a series of long non-coding RNAs (lncRNAs) which are characteristically more than 200 nucleotides long and can be involved in epigenetic modifications of DNA, and regulation of gene expression at transcriptional and post-transcriptional levels (103–105). The first lncRNA inside the PWS region is the Prader-Willi Region Non-Protein Coding RNA 1 (*PWRN1*), biallelically expressed in the testis and kidneys, and monoallelic expressed in the brain, in addition to being an alternative 5′ part of SNURF–SNRPN (106). Wawrzik et al. (107) hypothesized that the action of *PWRN1* on the imprinting mechanism may be indirect through keeping the paternal allele in an open chromatin configuration, allowing access to transcription factors (107). The main limitation for further confirmation studies is the lack of gene orthology in mice (108–110).

The Nuclear Pore Associated Protein 1 (*NPAP1*), formerly known as Chromosome 15 Open Reading Frame 2 (C15orf2), is an intronless gene that is biallelically expressed in adult testis and monoallelically expressed in fetal brain, including the hypothalamus which is related to several endocrine features of PWS (106, 111). Moreover, this gene is associated with the Nuclear Pore Complex (NPC), in which the main function is to regulate macromolecular transport between the nucleus and the cytoplasm. NPCs also participates in several nuclear processes, such as gene regulation, mRNA biogenesis, and cell cycle control. Likewise *PWRN1*, due to the lack of orthology in mice the exact role of the *NPAP1* gene in the development of PWS is not clear (112).

The Small Nuclear Ribonucleoprotein Polypeptide N (*SNRPN*) gene is located within the central region associated with PWS and has an important regulatory role over the imprinted genes located in chromosome 15 (113, 114), while the SNRPN Upstream Reading Frame (*SNURF*) gene is encoded by an evolutionarily-conserved upstream open reading frame and is localized to the nucleus (115). *SNURF-SNRPN* is a complex bicistronic gene encoding two different proteins, and the PWS-IC is found at its 5' end. *SNURF* is encoded by exons 1–3 and produces a small nuclear protein of unknown function (113), exons 4–10 correspond to the *SNRPN* portion and encode the protein SmN, involved in mRNA splicing (43). It also holds six snoRNA genes located telomerically which are expressed as a long transcript (46). The SmN protein shows the highest expression in the brain and heart (115–117). Despite its central position in PWS, the function and regulation of the many alternative transcripts of *SNURF-SNRPN* are still poorly understood (48).

Within the long *SNURF-SNRPN* transcript, there are a series of Small Nucleolar RNAs (snoRNAs) thought to participate in DNA methylation, alternative splicing and post-transcriptional regulation (10, 118). The PWS region encompasses five single copy snoRNA genes (*SNORD64, SNORD107, SNORD108, SNORD109A*, and *SNORD109B*) and two snoRNA gene clusters (*SNORD115* and *SNORD116*). The expression of SNORD genes varies in different human and mouse tissues, suggesting specificity in post-transcriptional activity (46, 119–122). Although most of the SNORDs are ubiquitously expressed in human tissues, *SNORD115* and *SNORD109B* appear to be restricted to the brain. Our understanding of the single-copy SNORDs in PWS remains extremely limited, but some progress has been made with the clusters: *SNORD116* has 29 tandemly repeats and *SNORD115* is composed of 48 gene copies (118). Given that SNORD sequences are well-conserved across placental mammals (especially in primates and rodents), this suggests they have an evolutionary functional role (123, 124).

A minimal critical region has emerged implicating that the *SNORD116* cluster is crucial for most of the PWS phenotype, based on clinical evidence on rare patients with small deletions (150–200 Kb) or translocations (11, 125–128). Experimental studies on *Snord116*-KO mice displayed PWS features such as post-natal growth retardation and hyperphagia (129–132). Remarkably, a *Snord116*-KO mice model specifically in NPY neurons in the hypothalamic arcuate nucleus summarized the same overall phenotype observed in mice lacking *Snord116* globally; low birth weight, increased body weight gain in early adulthood, increased energy expenditure and hyperphagia (130). This suggests an important role of *Snord116* in controlling NPY neuronal functions, and thus food intake and energy homeostasis. Also, a recent study reported *Snord116*-deficient mice with decreased activity of the hypothalamic prohormone convertase PC1 impairing the prohormone processing of proinsulin, pro-GH-releasing hormone, and proghrelin, pointing to an important part of *SNORD116* and PC1 deficiency in the main neuroendocrine features of PWS (133). Interestingly, it was shown that a mouse *Snord116* deletion model displayed loss or shift in methylation dynamics in 97% of CpG islands in the cerebral cortex dependent on the circadian cycle. And this disrupted epigenetic rhythm had a strong overlap between mouse and human genes related to meal timing, circadian biology, and obesity (134). In the recent years, *SNORD116* has emerged as a critical, and possibly, determinant candidate in PWS not only by its highly conserved sequence in the minimal critical region, but also because paternal deletions affecting the expression of *NDN, MKRN3, MAGEL2*, or *SNORD115* genes do not address the full spectrum of PWS symptoms (10, 100, 123, 135, 136).

The Imprinted in Prader-Willi Syndrome (*IPW*) gene is a lncRNA known to modulate another evolutionarily distinct imprinted gene cluster at the human chromosomal region 14q32 expressed only from maternally inherited alleles (137). *IPW* is widely expressed both in fetal and adult tissues, exclusively from the paternal allele (138). It has been postulated that *IPW* has no biological consequences in PWS, based on the relatively poor conservation between human and mouse sequences (138), and the fact that mice with a paternally inherited deletion including *Ipw* did not show PWS symptoms (139). However, Stelzer et al. (137) proposed that lack of expression of *IPW* results in aberrant upregulation of maternally expressed genes at the 14q32 imprinted cluster, pointing that the action of *IPW* on the imprinting mechanism of this locus occurs by histone modification, and consequently, transcription reduction (137). This hypothesis is supported by clinical reports of affected individuals with mUPD 14 (overexpression of maternal genes) presenting PWS-like phenotypes, such as neonatal hypotonia, small hands and feet, intellectual disability and hyperphagia (140–142). These findings pinpoint a regulatory cross-talk between 15q11-13 and 14q32 imprinted loci, but further, suggest that some PWS phenotypes may arise from different chromosomal regions other than the PWS critical locus (143, 144).

*SNORD115* gene is the most characterized SNORD within the PWS region. It presents a complementary sequence of 18 nucleotides with the mRNA encoding the serotonin receptor 5-HT2C, perfectly base pairing with exon V that undergoes both alternative RNA splicing and RNA editing (post-transcriptional changes to specific nucleotide sequences) (118). Mice with a large deletion encompassing the *Snord115* cluster developed normally to adulthood with apparently no significant defects (139). And there are also clinical reports on patients with an entire deletion of the *SNORD115* gene cluster that did not present any PWS major clinical signs (135, 136). Taken together, these findings suggest that lack of *SNORD115* is not sufficient to cause PWS, but a phenotypic effect when absent along with other genes in the PWS critical region cannot be excluded. Actually, the *5-HT2C* gene encodes G protein-coupled receptor specific to the brain, whose activation is associated with a variety of physiological processes, such as dopamine modulation, anxiety, sleep regulation, satiety response, energy balance, and locomotor activity (145). Interestingly, experimental studies have described 5-HT2C receptor knockout mice that developed are hyperphagia and late-onset obesity, two major clinical features of PWS in humans (146, 147). Therefore, the absence of *SNORD115* expression in PWS accompanied by the possible post-transcriptional impairment of the 5-HT2C receptor activity may be partly responsible for some of the behavioral and metabolic features of the syndrome.

The establishment of a causal genotype-phenotype relationship can bring light to new therapeutic approaches for PWS. Epigenetic therapy has been used in cancer treatment mostly focusing on the identification of small molecules and compounds with the capacity to reverse the epigenetic changes (epigenome reprogramming) (145, 148). The successful experience obtained from the epigenetic-cancer therapies contributes to the development of similar approaches for genomic imprinting disorders. Recent studies have shown that histone methyltransferase inhibitors are capable of reactivating the expression of paternally expressed SNRPN and *SNORD116* from the maternal chromosome, both in PWS mouse models and in cultured PWS patient-derived fibroblasts (149, 150). Although further investigation needs to be performed *in vivo*, epigenetic therapy aiming PWS genes in the maternal chromosome could reverse, or at least regulate, some PWS clinical conditions such as hyperphagia and behavioral problems (151). This data supports future studies to assess translational epigenetic-based therapies for PWS in humans.

## Conclusion

PWS is a complex imprinting disorder caused by the lack of expression of paternally-inherited genes on chromosome 15q11-q13 with severe implications on endocrine, cognitive and neurologic systems, metabolism, and behavior. The PWS critical region encompasses five protein-coding genes (*MKRN3, MAGEL2, NDN, NPAP1*, and *SNURF-SNRPN*) and more than 80 RNA genes (*PWRN1, IPW*, and several SNORDs) but their contribution to unique PWS phenotypes is still unclear. The broad clinical spectrum and the absence of a clear genotype-phenotype specific correlation imply that the numerous genes involved in PWS have an additive deleterious effect when deficiently expressed. So far, the lack of expression of the *SNORD116* gene cluster has arisen as the best explanation for most of the PWS phenotype, yet there is a clear need to investigate more of its mechanism of action, especially the incorporation into ribonucleoprotein complexes, possibly acting in hypothalamic and endocrine functions in adulthood and perinatal period. Besides *SNORD115* and *SNORD116*, our understanding of the biology of the PWS SNORD genes is still rather shallow. These SNORDs exhibit all the hallmarks of RNA methylation guides and can associate with other proteins to form functional ribonucleoprotein complexes. Also, the SNORD sequences are well-conserved across placental mammals, strongly asserting that they have a functional role as RNA entities under evolutionary pressure. A better understanding about genotype-phenotype in PWS can open space for new therapeutic approaches especially for patients that present side effects related to the current standard treatment, and develop genetic counseling for the different levels of severity in PWS that require specific and constant medical follow-up, improving the life quality of patients, family, and caretakers.

## Author Contributions

RC, IF, HC, and LGu contributed to the writing of the manuscript. LGo provided consultation and contributed to the writing of the manuscript.

### Conflict of Interest

The authors declare that the research was conducted in the absence of any commercial or financial relationships that could be construed as a potential conflict of interest.

## References

[1] Prader A Labhart A Willi H Ein syndrom von adipositas, kleinwuchs, kryptorchismus und oligophrenie nach myatonieartigem zustand im neugeborenenalter. Schweiz Med Wochenschr. (1956) 86:1260–1.

[2] Butler MG Meaney FJ Palmer CG Opitz JM Reynolds JF. Clinical and cytogenetic survey of 39 individuals with Prader-Labhart-Willi syndrome. Am J Med Genet. (1986) 23:793–809. 10.1002/ajmg.1320230307 3953677 PMC5494992

[3] Nicholls RD Knoll JH Butler MG Karam S Lalande M. Genetic imprinting suggested by maternal heterodisomy in nondeletion Prader-Willi syndrome. Nature. (1989) 342:281–5. 10.1038/342281a0 2812027 PMC6706849

[4] Butler MG. Genomic imprinting disorders in humans: a mini-review. J Assist Reprod Genet. (2009) 26:477–86. 10.1007/s10815-009-9353-3 19844787 PMC2788689

[5] Driscoll DJ Miller JL Schwartz S Cassidy SB Prader-Willi Syndrome. In: Adam MP Ardinger HH Pagon RA Wallace SE Bean LJH Stephens K, editors. GeneReviews. Seattle, WA: University of Washington (2017). p. 1–38.

[6] Whittington JE Butler JV Holland AJ. Changing rates of genetic subtypes of Prader-Willi syndrome in the UK. Eur J Hum Genet. (2007) 15:127–30. 10.1038/sj.ejhg.5201716 16957680

[7] Cassidy SB Driscoll DJ. Prader–Willi syndrome. Eur J Hum Genet. (2009) 17:3–13. 10.1038/ejhg.2008.165 18781185 PMC2985966

[8] Nicholls RD Knepper JL. Genome organization, function, and imprinting in Prader-Willi and Angelman syndromes. Annu Rev Genomics Hum Genet. (2001) 2:153–75. 10.1146/annurev.genom.2.1.153 11701647

[9] Angulo MA Butler MG Cataletto ME. Prader-Willi syndrome: a review of clinical, genetic, and endocrine findings. J Endocrinol Invest. (2015) 38:1249–63. 10.1007/s40618-015-0312-9 26062517 PMC4630255

[10] Cheon CK. Genetics of Prader-Willi syndrome and Prader-Will-Like syndrome. Ann Pediatr Endocrinol Metab. (2016) 21:126–35. 10.6065/apem.2016.21.3.126 27777904 PMC5073158

[11] Buiting K Prader-Willi syndrome and Angelman syndrome. Am J Med Genet Part C Semin Med Genet. (2010) 154:365–76. 10.1002/ajmg.c.30273 20803659

[12] Chamberlain SJ Lalande M Neurobiology of disease neurodevelopmental disorders involving genomic imprinting at human chromosome 15q11 – q13. Neurobiol Dis. (2010) 39:13–20. 10.1016/j.nbd.2010.03.011 20304067

[13] Gunay-Aygun M Schwartz S Heeger S O'Riordan MA Cassidy SB. The changing purpose of Prader-Willi syndrome clinical diagnostic criteria and proposed revised criteria. Pediatrics. (2001) 108:e92–e92. 10.1542/peds.108.5.e92 11694676

[14] Miller JL Lynn CH Driscoll DC Goldstone AP Gold JA Kimonis V Nutritional phases in Prader-Willi syndrome. Am J Med Genet Part A. (2011) 155:1040–9. 10.1002/ajmg.a.33951 PMC3285445 21465655

[15] Butler MG Sturich J Myers SE Gold J Kimonis V Driscoll DJ. Is gestation in Prader-Willi syndrome affected by the genetic subtype? J Assist Reprod Genet. (2009) 26:461–6. 10.1007/s10815-009-9341-7 19760168 PMC2767487

[16] Cassidy SB Schwartz S Miller JL Driscoll DJ Prader-Willi syndrome. Genet Med. (2012) 14:10–26. 10.1038/gim.0b013e31822bead0 22237428

[17] Richer LP Shevell MI Miller SP. Diagnostic profile of neonatal hypotonia: an 11-year study. Pediatr Neurol. (2001) 25:32–7. 10.1016/S0887-8994(01)00277-6 11483393

[18] Whittington J Holland A Webb T Butler J Clarke D Boer H. Academic underachievement by people with Prader-Willi syndrome. J Intellect Disabil Res. (2004) 48:188–200. 10.1111/j.1365-2788.2004.00473.x 14723660

[19] Jauregi J Arias C Vegas O Alén F Martinez S Copet P. A neuropsychological assessment of frontal cognitive functions in Prader-Willi syndrome. J Intellect Disabil Res. (2007) 51:350–65. 10.1111/j.1365-2788.2006.00883.x 17391252

[20] Copet P Jauregi J Laurier V Ehlinger V Arnaud C Cobo AM. Cognitive profile in a large french cohort of adults with Prader-Willi syndrome: differences between genotypes. J Intellect Disabil Res. (2010) 54:204–15. 10.1111/j.1365-2788.2010.01251.x 20136683

[21] Gillett E Perez I. Disorders of sleep and ventilatory control in Prader-Willi Syndrome. Diseases. (2016) 4:23. 10.3390/diseases4030023 28933403 PMC5456282

[22] Brambilla P Crinò A Bedogni G Bosio L Cappa M Corrias A. Metabolic syndrome in children with Prader-Willi syndrome: the effect of obesity. Nutr Metab Cardiovasc Dis. (2011) 21:269–76. 10.1016/j.numecd.2009.10.004 20089384

[23] Crinò A Fintini D Bocchini S Grugni G Diabetes, metabolic syndrome and obesity: targets and therapy dovepress obesity management in Prader-willi syndrome: current perspectives. Diabetes Metab Syndr Obes Targets Ther. (2018) 11:579–93. 10.2147/DMSO.S141352 PMC6175547 30323638

[24] Butler JV Whittington JE Holland AJ Boer H Clarke D Webb T. Prevalence of, and risk factors for, physical ill-health in people with Prader-Willi syndrome: a population-based study. Dev Med Child Neurol. (2002) 44:248–55. 10.1017/S001216220100202X 11995893

[25] Whittington JE Holland AJ Webb T. Ageing in people with Prader-Willi syndrome: mortality in the UK population cohort and morbidity in an older sample of adults. Psychol Med. (2015) 45:615–21. 10.1017/S0033291714001755 25088280

[26] Tauber M Cutfield W. KIGS highlights: growth hormone treatment in Prader-Willi Syndrome. Horm Res. (2007) 68(Suppl 5):48–50. 10.1159/000110475 18174707

[27] Grugni G Sartorio A Crinò A. Growth hormone therapy for Prader–Willi syndrome: challenges and solutions. Ther Clin Risk Manag. (2016) 12:873–81. 10.2147/TCRM.S70068 27330297 PMC4898426

[28] Lindgren AC. Somatropin therapy for children with Prader-Willi syndrome: guidelines for use. Treat Endocrinol. (2006) 5:223–8. 10.2165/00024677-200605040-00003 16879001

[29] Höybye C. Five-years growth hormone (GH) treatment in adults with Prader-Willi syndrome. Acta Paediatr. (2007) 96:410–3. 10.1111/j.1651-2227.2006.00051.x 17407467

[30] Mogul HR Lee PDK Whitman BY Zipf WB Frey M Myers S. Growth hormone treatment of adults with Prader-Willi syndrome and growth hormone deficiency improves lean body mass, fractional body fat, and serum triiodothyronine without glucose impairment: results from the United States multicenter trial. J Clin Endocrinol Metab. (2008) 93:1238–45. 10.1210/jc.2007-2212 18211968

[31] Siemensma EPC Tummers-de Lind van Wijngaarden RFA Festen DAM Troeman ZCE van Alfen-van der Velden AAEM Otten BJ. Beneficial effects of growth hormone treatment on cognition in children with Prader-Willi syndrome: a randomized controlled trial and longitudinal study. J Clin Endocrinol Metab. (2012) 97:2307–14. 10.1210/jc.2012-1182 22508707

[32] Berini J Spica Russotto V Castelnuovo P Di Candia S Gargantini L Grugni G. Growth hormone therapy and respiratory disorders: long-term follow-up in PWS children. J Clin Endocrinol Metab. (2013) 98:E1516–23. 10.1210/jc.2013-1831 23894156

[33] Deal CL Tony M Höybye C Allen DB Tauber M Christiansen JS. Growth hormone research society workshop summary: consensus guidelines for recombinant human growth hormone therapy in Prader-Willi syndrome. J Clin Endocrinol Metab. (2013) 98:E1072–87. 10.1210/jc.2012-3888 23543664 PMC3789886

[34] Dykens EM Roof E Hunt-Hawkins H. Cognitive and adaptive advantages of growth hormone treatment in children with Prader-Willi syndrome. J Child Psychol Psychiatry Allied Discip. (2017) 58:64–74. 10.1111/jcpp.12601 27481444 PMC5161611

[35] Crinò A Schiaffini R Ciampalini P Spera S Beccaria L Benzi F. Hypogonadism and pubertal development in Prader-Willi syndrome. Eur J Pediatr. (2003) 162:327–33. 10.1007/s00431-002-1132-4 12692714

[36] Heksch R Kamboj M Anglin K Obrynba K. Review of Prader-Willi syndrome: the endocrine approach. Transl Pediatr. (2017) 6:274–85. 10.21037/tp.2017.09.04 29184809 PMC5682385

[37] Muscogiuri G Formoso G Pugliese G Ruggeri RM Scarano E Colao A. Prader- Willi syndrome: an uptodate on endocrine and metabolic complications. Rev Endocr Metab Disord. (2019) 20:239–50. 10.1007/s11154-019-09502-2 31065942

[38] Tauber M Barbeau C Jouret B Pienkowski C Malzac P Moncla A. Auxological and endocrine evolution of 28 children with Prader-Willi syndrome: effect of GH therapy in 14 children. Horm Res Paediatr. (2000) 53:279–87. 10.1159/000053184 11146368

[39] Corrias A Grugni G Crinò A Di Candia S Chiabotto P Cogliardi A. Assessment of central adrenal insufficiency in children and adolescents with Prader-Willi syndrome. Clin Endocrinol. (2012) 76:843–50. 10.1111/j.1365-2265.2011.04313.x 22150958

[40] Beauloye V Dhondt K Buysse W Nyakasane A Zech F De Schepper J Evaluation of the hypothalamic-pituitary-adrenal axis and its relationship with central respiratory dysfunction in children with Prader-Willi syndrome rare endocrinological diseases. Orphanet J Rare Dis. (2015) 10:4–11. 10.1186/s13023-015-0312-z 26329144 PMC4557896

[41] Grugni G Beccaria L Corrias A Crinò A Cappa M De Medici C. Central adrenal insufficiency in young adults with Prader-Willi Syndrome. Clin Endocrinol. (2013) 79:371–8. 10.1111/cen.12150 23311724

[42] Smith A Hung D The dilemma of diagnostic testing for Prader-Willi syndrome. Transl Pediatr. (2017) 5:46–56. 10.21037/tp.2016.07.04 PMC5253259 28164030

[43] Glenn CC Saitoh S Jong MTC Filbrandt MM Surti U Driscoll DJ. Gene structure, DNA methylation, and imprinted expression of the human SNRPN gene. Am J Hum Genet. (1996) 58:335–46. 8571960 PMC1914536

[44] Ohta T Gray TA Rogan PK Buiting K Gabriel JM Saitoh S. Imprinting-mutation mechanisms in Prader-Willi Syndrome. AGHG. (1999) 64:397–413. 10.1086/302233 9973278 PMC1377750

[45] Yazdi PG Su H Ghimbovschi S Fan W Coskun PE Nalbandian A. Differential gene expression reveals mitochondrial dysfunction in an imprinting center deletion mouse model of prader-willi syndrome. Clin Transl Sci. (2013) 6:347–55. 10.1111/cts.12083 24127921 PMC3815468

[46] Galiveti CR Raabe CA Konthur Z Rozhdestvensky TS. Differential regulation of non-protein coding RNAs from Prader-Willi Syndrome locus. Sci Rep. (2014) 4:6445. 10.1038/srep06445 25246219 PMC4171697

[47] Butler MG Wang K Marshall JD Naggert JK Rethmeyer JA Gunewardena SS Coding and noncoding expression patterns associated with rare obesity-related disorders: Prader-Willi and Alstrom syndromes. Adv Genomics Genet. (2015) 50:53–75. 10.2147/AGG.S74598 PMC4334166 25705109

[48] Koufaris C Alexandrou A Papaevripidou I Alexandrou I Christophidou-Anastasiadou V Sismani C. Deletion of SNURF/SNRPN U1B and U1B^*^ upstream exons in a child with developmental delay and excessive weight. J Genet. (2016) 95:621–4. 10.1007/s12041-016-0666-6 27659333

[49] Bittel DC Butler MG. Prader-Willi syndrome: clinical genetics, cytogenetics and molecular biology. Expert Rev Mol Med. (2005) 7:1–20. 10.1017/S1462399405009531 16038620 PMC6750281

[50] Christian SL Robinson WP Huang B Mutirangura A Line MR Nakao M. Molecular characterization of two proximal deletion breakpoint regions in both Prader-Willi and Angelman syndrome patients. Am J Hum Genet. (1995) 57:40–8. 7611294 PMC1801233

[51] Hartin SN Hossain WA Francis D Godler DE Barkataki S Butler MG. Analysis of the Prader–Willi syndrome imprinting center using droplet digital PCR and next-generation whole-exome sequencing. Mol Genet Genomic Med. (2019) 7:1–10. 10.1002/mgg3.575 30793526 PMC6465664

[52] Glenn CC Driscoll DJ Yang TP Nicholls RD. Genomic imprinting: potential function and mechanisms revealed by the Prader-Willi and Angelman syndromes. Mol Hum Reprod. (1997) 3:321–32. 10.1093/molehr/3.4.321 9237260

[53] Kosaki K McGinniss MJ Veraksa AN McGinnis WJ Jones KL. Prader-Willi and Angelman syndromes: diagnosis with a bisulfite-treated methylation-specific PCR method. Am J Med Genet. (1997) 73:308–13. 10.1002/(SICI)1096-8628(19971219)73:3<308::AID-AJMG15>3.0.CO;2-N 9415690

[54] Botezatu A Puiu M Cucu N Diaconu CC Badiu C Arsene C. Comparative molecular approaches in Prader-Willi syndrome diagnosis. Gene. (2016) 575:353–8. 10.1016/j.gene.2015.08.058 26335514

[55] Bittel DC Kibiryeva N Butler MG. Methylation-specific multiplex ligation-dependent probe amplification analysis of subjects with chromosome 15 abnormalities. Genet Test. (2007) 11:467–76. 10.1089/gte.2007.0061 18294067 PMC5494700

[56] Henkhaus RS Kim SJ Kimonis VE Gold J-A Dykens EM Driscoll DJ. Methylation-specific multiplex ligation-dependent probe amplification and identification of deletion genetic subtypes in Prader-Willi syndrome. Genet Test Mol Biomarkers. (2011) 16:178–86. 10.1089/gtmb.2011.0115 21977908 PMC3306590

[57] White HE Hall VJ Cross NCP. Methylation-sensitive high-resolution melting-curve analysis of the SNRPN gene as a diagnostic screen for Prader-Willi and Angelman syndromes. Clin Chem. (2007) 53:1960–2. 10.1373/clinchem.2007.093351 17890436

[58] Ferreira IR Darleans dos Santos Cunha W Henrique Ferreira Gomes L Azevedo Cintra H Lopes Cabral Guimarães Fonseca L Ferreira Bastos E A rapid and accurate methylation-sensitive high-resolution melting analysis assay for the diagnosis of Prader Willi and Angelman patients. Mol Genet Genomic Med. (2019) 7:1–10. 10.1002/mgg3.637 31033246 PMC6565559

[59] Hawkey CJ Smithies A. The Prader-Willi syndrome with a 15/15 translocation. Case report and review of the literature. J Med Genet. (1996) 13:152–7. 10.1136/jmg.13.2.152 933113 PMC1013377

[60] Smith A Lindeman R Volpato F Kearney A White S Haan E. A *de novo* unbalanced reciprocal translocation identified as paternal in origin in the Prader-Willi syndrome. Hum Genet. (1991) 86:534–6. 10.1007/BF00194651 2016095

[61] Robinson WP Dutly F Nicholls RD Bernasconi F Pefiaherrera M Michaelis RC. The mechanisms involved in formation of deletions. J Med Genet. (1998) 35:130–6. 10.1136/jmg.35.2.130 9580159 PMC1051217

[62] Kuslich CD Kobori JA Mohapatra G Gregorio-King C Donlon TA. Prader-Willi syndrome is caused by disruption of the SNRPN gene. Am J Hum Genet. (1999) 64:70–6. 10.1086/302177 9915945 PMC1377704

[63] Flori E Biancalana V Girard-Lemaire F Favre R Flori J Doray B Difficulties of genetic counselling and prenatal diagnosis in a consanguineous couple segregating for the same translocation (14;15) (q11;q13) and at risk for Prader-Willi and Angelman syndromes. Eur J Hum Genet. (2004) 12:181–6. 10.1038/sj.ejhg.5201134 14694357

[64] Kim SJ Miller JL Kuipers PJ German JR Beaudet AL Sahoo T. Unique and atypical deletions in Prader-Willi syndrome reveal distinct phenotypes. Eur J Hum Genet. (2012) 20:283–90. 10.1038/ejhg.2011.187 22045295 PMC3283188

[65] Yip MY. Uniparental disomy in Robertsonian translocations: strategies for uniparental disomy testing. Transl Pediatr. (2014) 3:98–107. 10.3978/j.issn.2224-4336.2014.03.03 26835328 PMC4729106

[66] Santoro SL Hashimoto S McKinney A Mihalic Mosher T Pyatt R Reshmi SC. Assessing the clinical utility of SNP microarray for Prader-Willi syndrome due to uniparental disomy. Cytogenet Genome Res. (2017) 152:105–9. 10.1159/000478921 28746920

[67] Beygo J Buiting K Ramsden SC Ellis R Clayton-Smith J Kanber D Update of the EMQN/ACGS best practice guidelines for molecular analysis of Prader-Willi and Angelman syndromes. Eur J Hum Genet. (2019) 15:1326–40. 10.1038/s41431-019-0435-0 PMC6777528 31235867

[68] Torrado M Araoz V Baialardo E Abraldes K Mazza C Krochik G. Clinical-etiologic correlation in children with Prader-Willi syndrome (PWS): an interdisciplinary study. Am J Med Genet Part A. (2007) 143A:460–8. 10.1002/ajmg.a.31520 17163531

[69] Butler MG Bittel DC Bittel N Talebizadeh KZ Thompson T. Behavioral differences among subjects with Prader-Willi Syndrome and type I or type II deletion and maternal disomy. Pediatrics. (2004) 113:565–73. 10.1542/peds.113.3.565 14993551 PMC6743499

[70] Hartley SL MacLean WE Butler MG Zarcone J Thompson T Maladaptive behaviors and risk factors among the genetic subtypes of Prader-Willi syndrome. Am J Med Genet Part A. (2005) 136A:140–5. 10.1002/ajmg.a.30771 PMC1896317 15940679

[71] Descheemaeker MJ Govers V Vermeulen P Fryns JP Pervasive developmental disorders in Prader–Willi syndrome: the Leuven experience in 59 subjects and controls. Am J Med Genet Part A. (2006) 140A:1136–42. 10.1002/ajmg.a.31235 16646032

[72] Dykens EM. Are Jigsaw Puzzle skills ‘spared' in persons with Prader-Willi syndrome? J Child Psychol Psychiatry. (2002) 43:343–52. 10.1111/1469-7610.00025 11944876

[73] Whittington J Holland A Webb T Butler J Clarke D Boer H. Cognitive abilities and genotype in a population-based sample of people with Prader-Willi syndrome. J Intellect Disabil Res. (2004) 48:172–87. 10.1111/j.1365-2788.2004.00556.x 14723659

[74] Yang L Zhan G Ding J Wang H Ma D Huang G. Psychiatric illness and intellectual disability in the Prader–Willi syndrome with different molecular defects - a meta analysis. PLoS ONE. (2013) 8:e72640. 10.1371/journal.pone.0072640 23967326 PMC3743792

[75] Dykens EM Roof E Hunt-Hawkins H Dankner N Lee EB Shivers CM. Diagnoses and characteristics of autism spectrum disorders in children with Prader-Willi syndrome. J Neurodev Disord. (2017) 9:18. 10.1186/s11689-017-9200-2 28592997 PMC5458479

[76] Jong MTC Gray TA Ji Y Glenn CC Saitoh S Driscoll DJ. A novel imprinted gene, encoding a RING Zinc-finger protein, and overlapping antisense transcript in the Prader-Willi syndrome critical region. Hum Mol Genet. (1999) 8:783–93. 10.1093/hmg/8.5.783 10196367

[77] Valadares LP Meireles CG De Toledo IP Santarem de Oliveira R Gonçalves de Castro LC Abreu AP. *MKRN3* mutations in central precocious puberty: a systematic review and meta-analysis. J Endocr Soc. (2019) 3:979–95. 10.1210/js.2019-00041 31041429 PMC6483926

[78] Abreu AP Delanie BM Brito VN Kaiser UB Latronico AC. A new pathway in the control of the initiation of puberty: the *MKRN3* gene. J Mol Endocrinol. (2015) 54:R131–9. 10.1530/JME-14-0315 25957321 PMC4573396

[79] Macedo DB Abreu AP Reis ACS Montenegro LR Dauber A Beneduzzi D. Central precocious puberty that appears to be sporadic caused by paternally inherited mutations in the imprinted gene makorin ring finger 3. J Clin Endocrinol Metab. (2014) 99:E1097–103. 10.1210/jc.2013-3126 24628548 PMC4037732

[80] Schreiner F Gohlke B Hamm M Korsch E Woelfle J. *MKRN3* mutations in familial central precocious puberty. Horm Res Paediatr. (2014) 82:122–6. 10.1159/000362815 25011910

[81] Settas N Dacou-Voutetakis C Karantza M Kanaka-Gantenbein C Chrousos GP Voutetakis A. Central precocious puberty in a girl and early puberty in her brother caused by a novel mutation in the *MKRN3* gene. J Clin Endocrinol Metab. (2014) 99:E647–51. 10.1210/jc.2013-4084 24438377

[82] Schaaf CP Gonzalez-Garay ML Xia F Potocki L Gripp KW Zhang B. Truncating mutations of *MAGEL2* cause Prader-Willi phenotypes and autism. Nat Genet. (2013) 45:1405–8. 10.1038/ng.2776 24076603 PMC3819162

[83] Bischof JM Stewart LC Wevrick R. Inactivation of the mouse *MAGEL2* gene results in growth abnormalities similar to Prader-Willi syndrome. Hum Mol Genet. (2007) 16:2713–9. 10.1093/hmg/ddm225 17728320

[84] Devos J Weselake SV Wevrick R. *MAGEL2*, a Prader-Willi syndrome candidate gene, modulates the activities of circadian rhythm proteins in cultured cells. J Circadian Rhythms. (2011) 9:12. 10.1186/1740-3391-9-12 22208286 PMC3278377

[85] Fliers E The human hypothalamus: basic and clinical aspects. J Neuroendocrinol. (2004) 16:1009–10. 10.1111/j.1365-2826.2005.01255.x

[86] Myers SE Davis A Whitman BY Santiago JV Landt M. Leptin concentrations in Prader-Willi syndrome before and after growth hormone replacement. Clin Endocrinol. (2000) 52:101–5. 10.1046/j.1365-2265.2000.00868.x 10651760

[87] Mercer RE Michaelson SD Chee MJS Atallah TA Wevrick R Colmers WF. *MAGEL2* is required for leptin-mediated depolarization of POMC neurons in the hypothalamic arcuate nucleus in mice. PLoS Genet. (2013) 9:e1003207. 10.1371/journal.pgen.1003207 23341784 PMC3547795

[88] Varela L Horvath TL. Leptin and insulin pathways in POMC and AgRP neurons that modulate energy balance and glucose homeostasis. EMBO Rep. (2012) 13:1079–86. 10.1038/embor.2012.174 23146889 PMC3512417

[89] Mercer RE Wevrick R. Loss of *MAGEL2*, a candidate gene for features of Prader-Willi syndrome, impairs reproductive function in mice. PLoS ONE. (2009) 4:e4291. 10.1371/journal.pone.0004291 19172181 PMC2627930

[90] Yoon H Enquist LW Dulac C. Olfactory inputs to hypothalamic neurons controlling reproduction and fertility. Cell. (2005) 123:669–82. 10.1016/j.cell.2005.08.039 16290037

[91] Fountain MD Schaaf CP. Prader-Willi syndrome and Schaaf-Yang syndrome: neurodevelopmental diseases intersecting at the *MAGEL2* gene. Diseases. (2016) 4:E2. 10.3390/diseases4010002 28933382 PMC5456300

[92] McCarthy J Lupo PJ Kovar E Rech M Bostwick B Scott D. Schaaf-Yang syndrome overview: report of 78 individuals. Am J Med Genet Part A. (2018) 176:2564–74. 10.1002/ajmg.a.40650 30302899 PMC6585857

[93] Miller NLG Wevrick R Mellon PL. Necdin, a Prader-Willi syndrome candidate gene, regulates gonadotropin-releasing hormone neurons during development. Hum Mol Genet. (2008) 18:248–60. 10.1093/hmg/ddn344 18930956 PMC2638776

[94] Jay P Rougeulle C Massacrier A Moncla A Mattel M Malzac P. The human necdin gene, *NDN*, is maternally imprinted and located in the Prader-Willi syndrome chromosomal region. Nat Genet. (1997) 17:357–61. 10.1038/ng1197-357 9354807

[95] Lee S Walker CL Karten B Kuny SL Tennese AA O'Neill MA. Essential role for the Prader–Willi syndrome protein necdin in axonal outgrowth. Hum Mol Genet. (2005) 14:627–37. 10.1093/hmg/ddi059 15649943

[96] Watrin F Roëckel N Lacroix L Mignon C Mattei MG Disteche C. The mouse Necdin gene is expressed from the paternal allele only and lies in the 7C region of the mouse chromosome 7, a region of conserved synteny to the human Prader-Willi syndrome region. Eur J Hum Genet. (1997) 5:324–32. 10.1159/000484784 9412790

[97] Miller J Silverstein J Shuster J Driscoll DJ Wagner M. Short-term effects of growth hormone on sleep abnormalities in Prader-Willi syndrome. J Clin Endocrinol Metab. (2006) 91:413–7. 10.1210/jc.2005-1279 16317059

[98] Muscatelli F Disruption of the mouse necdin gene results in hypothalamic and behavioral alteratio ns reminiscent of the human Prader-Willi syndrome. Hum Mol Genet. (2000) 9:3101–10. 10.1093/hmg/9.20.3101 11115855

[99] Zanella S Watrin F Mebarek S Marly F Roussel M Gire C. Necdin plays a role in the serotonergic modulation of the mouse respiratory network: implication for Prader-Willi syndrome. J Neurosci. (2008) 28:1745–55. 10.1523/JNEUROSCI.4334-07.2008 18272695 PMC6671529

[100] Kanber D Giltay J Wieczorek D Zogel C Hochstenbach R Caliebe A A paternal deletion of MKRN3, MAGEL2 and NDN does not result in Prader-Willi syndrome. Eur J Hum Genet. (2009) 17:582–90. 10.1038/ejhg.2008.232 19066619 PMC2986273

[101] Lee S. Expression and imprinting of *MAGEL2* suggest a role in Prader-Willi syndrome and the homologous murine imprinting phenotype. Hum Mol Genet. (2000) 9:1813–9. 10.1093/hmg/9.12.1813 10915770

[102] Pagliardini S Ren J Wevrick R Greer JJ. Developmental abnormalities of neuronal structure and function in prenatal mice lacking the Prader-Willi syndrome gene necdin. Am J Pathol. (2005) 167:175–91. 10.1016/S0002-9440(10)62964-1 15972963 PMC1603432

[103] Dhanoa JK Sethi RS Verma R Arora JS Mukhopadhyay CS. Long non-coding RNA: its evolutionary relics and biological implications in mammals: a review. J Anim Sci Technol. (2018) 60:25. 10.1186/s40781-018-0183-7 30386629 PMC6201556

[104] Fernandes J Acuña S Aoki J Floeter-Winter L Muxel S. Long non-coding RNA*s* in the regulation of gene expression: physiology and disease. Non-Coding RNA. (2019) 5:17. 10.3390/ncrna5010017 30781588 PMC6468922

[105] Mercer TR Dinger ME Mattick JS. Long non-coding RNAs: insights into functions. Nat Rev Genet. (2009) 10:155–9. 10.1038/nrg2521 19188922

[106] Buiting K Nazlican H Galetzka D Wawrzik M Groβ S Horsthemke B. C15orf2 and a novel noncoding transcript from the Prader–Willi/Angelman syndrome region show monoallelic expression in fetal brain. Genomics. (2007) 89:588–95. 10.1016/j.ygeno.2006.12.008 17337158

[107] Wawrzik M Spiess A-N Herrmann R Buiting K Horsthemke B. Expression of SNURF–SNRPN upstream transcripts and epigenetic regulatory genes during human spermatogenesis. Eur J Hum Genet. (2009) 17:1463–70. 10.1038/ejhg.2009.83 19471314 PMC2986690

[108] Chen Z Ju H Yu S Zhao T Jing X Li P. Prader–Willi region non-protein coding RNA 1 suppressed gastric cancer growth as a competing endogenous RNA of MiR-425-5p. Clin Sci. (2018) 132:1003–19. 10.1042/CS20171588 29535266

[109] Kung JTY Colognori D Lee JT. Long noncoding RNAs: past, present, and future. Genetics. (2013) 193:651–69. 10.1534/genetics.112.146704 23463798 PMC3583990

[110] Lee S Wevrick R. Identification of novel imprinted transcripts in the Prader-Willi syndrome and angelman syndrome deletion region: further evidence for regional imprinting control. Am J Hum Genet. (2000) 66:848–58. 10.1086/302817 10712201 PMC1288168

[111] Färber C Groß S Neesen J Buiting K Horsthemke B. Identification of a testis-specific gene (C15orf2) in the Prader–Willi syndrome region on chromosome 15. Genomics. (2000) 65:174–83. 10.1006/geno.2000.6158 10783265

[112] Neumann LC Markaki Y Mladenov E Hoffmann D Buiting K Horsthemke B. The imprinted *NPAP1*/C15orf2 gene in the Prader–Willi syndrome region encodes a nuclear pore complex associated protein. Hum Mol Genet. (2012) 21:4038–48. 10.1093/hmg/dds228 22694955

[113] Gray TA Saitoh S Nicholls RD. An imprinted, mammalian bicistronic transcript encodes two independent proteins. Proc Natl Acad Sci USA. (1999) 96:5616–21. 10.1073/pnas.96.10.5616 10318933 PMC21909

[114] Özçelik T Leff S Robinson W Donlon T Lalande M Sanjines E. Small nuclear ribonucleoprotein polypeptide N (SNRPN), an expressed gene in the Prader–Willi syndrome critical region. Nat Genet. (1992) 2:265–9. 10.1038/ng1292-265 1303277

[115] Rodriguez-Jato S Nicholls RD Driscoll DJ Yang TP. Characterization of cis- and trans-acting elements in the imprinted human *SNURF-SNRPN* locus. Nucleic Acids Res. (2005) 33:4740–53. 10.1093/nar/gki786 16116039 PMC1188517

[116] Cao Y AlHumaidi SS Faqeih EA Pitel BA Lundquist P Aypar U. A novel deletion of SNURF/SNRPN Exon 1 in a patient with Prader-Willi-like phenotype. Eur J Med Genet. (2017) 60:416–20. 10.1016/j.ejmg.2017.05.003 28554868

[117] Geuns E. Methylation imprints of the imprint control region of the SNRPN-gene in human gametes and preimplantation embryos. Hum Mol Genet. (2003) 12:2873–9. 10.1093/hmg/ddg315 14500540

[118] Cavaillé J Buiting K Kiefmann M Lalande M Brannan CI Horsthemke B. Identification of brain-specific and imprinted small nucleolar RNA genes exhibiting an unusual genomic organization. Proc Natl Acad Sci USA. (2000) 97:14311–6. 10.1073/pnas.250426397 11106375 PMC18915

[119] Castle JC Armour CD Löwer M Haynor D Biery M Bouzek H. Digital genome-wide NcRNA expression, including SnoRNAs, across 11 human tissues using polyA-neutral amplification. PLoS ONE. (2010) 5:e11779. 10.1371/journal.pone.0011779 20668672 PMC2909899

[120] Chamberlain SJ Chen PF Ng KY Bourgois-Rocha F Lemtiri-Chlieh F Levine ES. Induced pluripotent stem cell models of the genomic imprinting disorders angelman and Prader-Willi Syndromes. Proc Natl Acad Sci USA. (2010) 107:17668–73. 10.1073/pnas.1004487107 20876107 PMC2955112

[121] Martins-Taylor K Hsiao JS Chen P-F Glatt-Deeley H De Smith AJ Blakemore AIF. Imprinted expression of *UBE3A* in non-neuronal cells from a Prader–Willi syndrome patient with an atypical deletion. Hum Mol Genet. (2014) 23:2364–73. 10.1093/hmg/ddt628 24363065 PMC3976333

[122] Vitali P Royo H Marty V Bortolin-Cavaille M-L Cavaille J. Long nuclear-retained non-coding RNAs and allele-specific higher-order chromatin organization at imprinted snoRNA gene arrays. J Cell Sci. (2010) 123:70–83. 10.1242/jcs.054957 20016068

[123] Cavaillé J Box C/D small nucleolar RNA genes and the Prader-Willi syndrome: a complex interplay: box C/D SnoRNA genes and the Prader-Willi syndrome. Wiley Interdiscipl. Rev. RNA. (2017) 8:e1417 10.1002/wrna.1417 28296064

[124] Zhang YJ Yang JH Shi QS Zheng LL Liu J Zhou H. Rapid birth-and-death evolution of imprinted snoRNAs in the Prader-Willi syndrome locus: implications for neural development in *Euarchontoglires*. PLoS ONE. (2014) 9:e100329. 10.1371/journal.pone.0100329 24945811 PMC4063771

[125] Bieth E Eddiry E Gaston V Lorenzini F Buffet A Conte AF. Highly restricted deletion of the *SNORD116* region is implicated in Prader–Willi syndrome. Eur J Hum Genet. (2015) 23:252–5. 10.1038/ejhg.2014.103 24916642 PMC4297892

[126] Duker AL Ballif BC Bawle EV Person RE Mahadevan S Alliman S. Paternally inherited microdeletion at 15q11.2 confirms a significant role for the *SNORD116* C/D box SnoRNA cluster in Prader–Willi syndrome. Eur J Hum Genet. (2010) 18:1196–201. 10.1038/ejhg.2010.102 20588305 PMC2987474

[127] Sahoo T del Gaudio D German JR Shinawi M Peters SU Person RE. Prader-Willi phenotype caused by paternal deficiency for the HBII-85 C/D box small nucleolar RNA cluster. Nat Genet. (2008) 40:719–21. 10.1038/ng.158 18500341 PMC2705197

[128] de Smith AJ Purmann C Walters RG Ellis RJ Holder SE Van Haelst MM. A deletion of the HBII-85 class of small nucleolar RNAs (snoRNAs) is associated with hyperphagia, obesity and hypogonadism. Hum Mol Genet. (2009) 18:3257–65. 10.1093/hmg/ddp263 19498035 PMC2722987

[129] Ding F Li HH Zhang S Solomon NM Camper SA Cohen P. SnoRNA *SNORD116* (Pwcr1/MBII-85) deletion causes growth deficiency and hyperphagia in mice. PLoS ONE. (2008) 3:e1709. 10.1371/journal.pone.0001709 18320030 PMC2248623

[130] Qi Y Purtell L Fu M Lee NJ Aepler J Zhang L. *SNORD116* is critical in the regulation of food intake and body weight. Sci Rep. (2016) 6:18614. 10.1038/srep18614 26726071 PMC4698587

[131] Rozhdestvensky TS Robeck T Galiveti CR Raabe CA Seeger B Wolters A. Maternal transcription of non-protein coding RNAs from the PWS-critical region rescues growth retardation in mice. Sci Rep. (2016) 6:20398. 10.1038/srep20398 26848093 PMC4742849

[132] Skryabin BV Gubar LV Seeger B Pfeiffer J Handel S Robeck T. Deletion of the MBII-85 snoRNA gene cluster in mice results in postnatal growth retardation. PLoS Genet. (2007) 3:e235. 10.1371/journal.pgen.0030235 18166085 PMC2323313

[133] Burnett LC LeDuc CA Sulsona CR Paull D Rausch R Eddiry S. Deficiency in prohormone convertase PC1 impairs prohormone processing in Prader-Willi syndrome. J Clin Invest. (2017) 127:293–305. 10.1172/JCI88648 27941249 PMC5199710

[134] Coulson RL Yasui DH Dunaway KW Laufer BI Vogel Ciernia A Zhu Y. Snord116-dependent diurnal rhythm of DNA methylation in mouse cortex. Nat Commun. (2018) 9:1616. 10.1038/s41467-018-03676-0 29691382 PMC5915486

[135] Bürger J Horn D Tönnies H Neitzel H Reis A Familial interstitial 570 Kbp deletion of the *UBE3A* gene region causing angelman syndrome but not Prader-Willi syndrome: familial *UBE3A* gene deletion. Am J Med Genet. (2002) 111:233–7. 10.1002/ajmg.10498 12210318

[136] Runte M Varon R Horn D Horsthemke B Buiting K. Exclusion of the C/D box snoRNA gene cluster HBII-52 from a major role in Prader? Willi syndrome. Hum Genet. (2005) 116:228–30. 10.1007/s00439-004-1219-2 15565282

[137] Stelzer Y Sagi I Yanuka O Eiges R Benvenisty N. The noncoding RNA *IPW* regulates the imprinted DLK1-DIO3 locus in an induced pluripotent stem cell model of Prader-Willi syndrome. Nat Genet. (2014) 46:551–7. 10.1038/ng.2968 24816254

[138] Wevrick R Francke U. An imprinted mouse transcript homologous to the human imprinted in Prader-Willi syndrome (*IPW*) gene. Hum Mol Genet. (1997) 6:325–32. 10.1093/hmg/6.2.325 9063754

[139] Ding F Prints Y Dhar MS Johnson DK Montero CC Nicholls RD. Lack of Pwcr1/MBII-85 SnoRNA is critical for neonatal lethality in Prader–Willi syndrome mouse models. Mammalian Genome. (2005) 16:424–31. 10.1007/s00335-005-2460-2 16075369

[140] Falk MJ Curtis C Bass NE Zinn AB Schwartz S. Maternal uniparental disomy chromosome 14: case report and literature review. Pediatric Neurol. (2005) 32:116–20. 10.1016/j.pediatrneurol.2004.07.007 15664772

[141] Hordijk R Wierenga H Scheffer H Leegte B Hofstra R Stolte-Dijkstra I. Maternal uniparental disomy for chromosome 14 in a boy with a normal karyotype. J Med Genet. (1999) 36:782–5. 10.1136/jmg.36.10.782 10528860 PMC1734247

[142] Hosoki K Kagami M Tanaka T Kubota M Kurosawa K Kato M. Maternal Uniparental Disomy 14 Syndrome Demonstrates Prader-Willi Syndrome-Like Phenotype. J Pediatr. (2009) 155:900–03.e1. 10.1016/j.jpeds.2009.06.045 19800077

[143] Murrell A. Cross-talk between imprinted loci in Prader-Willi syndrome. Nat Genet. (2014) 46:528–30. 10.1038/ng.2994 24866188

[144] Patten MM Cowley M Oakey RJ Feil R. Regulatory links between imprinted genes: evolutionary predictions and consequences. Proc R Soc B Biol Sci. (2016) 283:20152760. 10.1098/rspb.2015.2760 26842569 PMC4760173

[145] Wold EA Wild CT Cunningham KA Zhou J. Targeting the 5-HT2C receptor in biological context and the current state of 5-HT2C receptor ligand development. Curr Top Med Chem. (2019) 19:1381–98. 10.2174/1568026619666190709101449 31288724 PMC6761005

[146] Nonogaki K Strack AM Dallman MF Tecott LH. Leptin-independent hyperphagia and type 2 diabetes in mice with a mutated serotonin 5-HT2C receptor gene. Nat Med. (1998) 4:1152–6. 10.1038/2647 9771748

[147] Tecott LH Sun LM Akana SF Strack AM Lowenstein DH Dallman MF. Eating disorder and epilepsy in mice lacking 5-HT2C serotonin receptors. Nature. (1995) 374:542–6. 10.1038/374542a0 7700379

[148] Biswas S Rao CM. Epigenetic tools (the writers, the readers and the erasers) and their implications in cancer therapy. Eur J Pharmacol. (2018) 837:8–24. 10.1016/j.ejphar.2018.08.021 30125562

[149] Kim Y Wang SE Jiang Y. Epigenetic therapy of Prader–Willi syndrome. Transl Res. (2019) 208:105–18. 10.1016/j.trsl.2019.02.012 30904443 PMC6527448

[150] Wang SE Jiang Y. Potential of epigenetic therapy for Prader-Willi syndrome. Trends Pharmacol Sci. (2019) 40:605–8. 10.1016/j.tips.2019.07.002 31353046

[151] Kim Y Lee H-M Xiong Y Sciaky N Hulbert SW Cao X. Targeting the histone methyltransferase G9a activates imprinted genes and improves survival of a mouse model of Prader–Willi syndrome. Nat Med. (2017) 23:213–22. 10.1038/nm.4257 28024084 PMC5589073

